# Experimental Proof of a Transformation Product Trap Effect with a Membrane Photocatalytic Process for VOC Removal

**DOI:** 10.3390/membranes12090900

**Published:** 2022-09-19

**Authors:** Fabien Gérardin, Julien Simard, Éric Favre

**Affiliations:** 1 Institut National de Recherche et de Sécurité, Rue du Morvan, CS60027, CEDEX, 54519 Vandœuvre, France; 2 Laboratoire Réactions et Génie des Procédés, UMR 7274 CNRS Université de Lorraine, 1 Rue Grandville BP20451, CEDEX, 54001 Nancy, France

**Keywords:** membrane separation, VOC removal, photocatalysis, transformation products, safety

## Abstract

The decomposition of volatile organic compounds by photocatalytic oxidation (PCO) has been widely studied. However, the technological development of this oxidative technique has to address how to handle the formation of transformation products. The work presented here investigates the original combination of a dense membrane separation process and PCO to intensify the trapping and reduction of PCO transformation products. Specific monitoring of toluene PCO transformation products, such as benzene and formaldehyde, in the outflow of both permeate and retentate compartments was proposed. The influence of operating parameters on the process, i.e., light intensity, pressure, membrane type, and catalyst mass, was also studied. The results reveal that membrane separation-PCO hybridization is particularly effective for reducing the presence of benzene and formaldehyde in the effluent treated. The benzene concentration in the outflow of the hybrid module can be reduced by a factor of 120 compared to that observed during the PCO of toluene alone.

## 1. Introduction

The problem of human exposure to volatile organic compounds (VOCs) is encountered in most confined spaces [1,2,3]. At both home and workplace, the presence of compounds, some of which are toxic to humans, is a major concern for health authorities [4,5,6]. The substances identified in indoor air are mainly related to intakes of outdoor air, building and furnishing materials, people’s activities, and cleaning products [7]. The compounds concerned include benzene, toluene, and formaldehyde [7]. Table 1 presents the typical concentrations observed in indoor air for these three compounds. The US Threshold Limit Value-Time-Weighted Averages [8] for toluene, benzene, and formaldehyde are 20 ppm, 0.5 ppm, and 100 ppb respectively.

**Figure 1:**
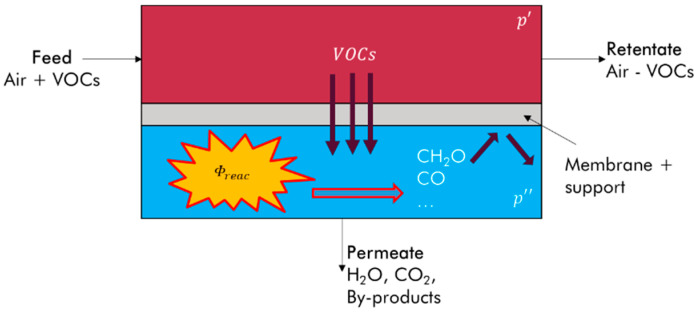
Principle of coupling of VOC permeation and photocatalytic reaction on the permeate compartment.

Moreover, the need to reduce the energy consumption of buildings has led to the limitation of their ventilation, thus increasing their indoor air concentration. The use of air treatment solutions may be appropriate when it is not possible to reduce the source of emissions of these toxic substances [12]. Photocatalytic oxidation (PCO) is well suited to the removal of low concentration VOCs [13,14,15,16,17,18]. While a number of photocatalytic materials have been scientifically investigated—such as ZnO, ZrO_2_, WO_3_, Ga_2_O_3_, and SnO_2_—titanium dioxide (TiO_2_) remains the material most frequently studied and implemented in commercialized systems [15,16,17,18,19,20,21]. The main advantages of this photocatalytic medium are that it is readily available, inexpensive, and can be activated by a light source in the UVA range for its anatase form [22]. However, PCO has been the subject of numerous studies showing that the mineralization of VOCs can result not only in the production of water and carbon dioxide, but can also simultaneously give rise to toxic transformation products (TPs) [23,24]. The literature indicates that, for example, the PCO of toluene leads to the formation of benzaldehyde, formaldehyde, acetaldehyde, and benzene, as well as to the deactivation of photocatalyst [13,25,26,27,28,29,30]. The pollution of photocatalyst in this case is mainly related to deposits of benzaldehyde on the active sites of the catalytic medium [31]. Most commercial air treatment units that use photocatalysis incorporate an adsorbent filter (activated carbon or molecular sieve) to limit the release of TPs into the environment [12]. The strategy of setting up a TP trapping stage with an adsorbent material is not a convincing solution. The risk of saturation of the adsorbent material over time and the emission of toxic compounds represents a hazard. An alternative approach based on a preliminary physical separation of the target pollutants from the air before their photocatalytic decomposition could effectively limit the presence of reaction intermediates and transformation products in the treated effluent. This decoupling effect between the feed flow and the treated air can be obtained by a membrane. The resulting principle of process hybridization is investigated in this paper. The objective is to extract VOCs from the air in a membrane module, so that the permeated compounds can be oxidized in the permeate compartment of the device (Figure 1). The photocatalytic medium is placed in the space of the permeate side and is not integrated in the membrane. The permeate photocatalysis configuration offers the opportunity in situ to generate a transmembrane driving force such as chemical pumping, leading to the separation of VOCs. Regarding gas permeation, combining membrane separation with PCO significantly increases the efficiency of the separation process [32]. A higher residence time in the permeate compartment should also lead to greater oxidation of the VOCs extracted. TPs are formed only in the permeate compartment but some membranes are more or less permeable to these TPs and these compounds can be observed in the retentate compartment. A dense membrane with relatively low permeability for any TPs should contribute to trapping these substances in the permeate compartment and limiting their transfer to the retentate compartment.

The aim of this study is to demonstrate through experiments that the combination of membrane separation and PCO described here makes it possible to limit the presence of undesirable TPs in the retentate that could be reused. The experiment was performed with an air/toluene mixture. Particular attention is focused on monitoring the evolution of the concentration of toluene PCO transformation products, such as benzene and formaldehyde, at the outlet of both the permeate and retentate compartments. Benzene and formaldehyde are compounds that are carcinogenic for humans and their concentration must be kept as low as possible in breathable air [4]. The toluene mineralization rate and the capacity of the photocatalyst to be regenerated according to the test cycles are also assessed. The impact of operating parameters on the process performances, i.e., light intensity, pressure, membrane type, and catalyst mass, are described and discussed. Based on an approach that was primarily experimental, the modeling of toluene removal is also presented in this study, opening the way for the rigorous design of a VOC removal unit with a minimal level of toxic transformation products in the air treated.

## 2. Materials and Method

### 2.1. Experiment

**Figure 2:**
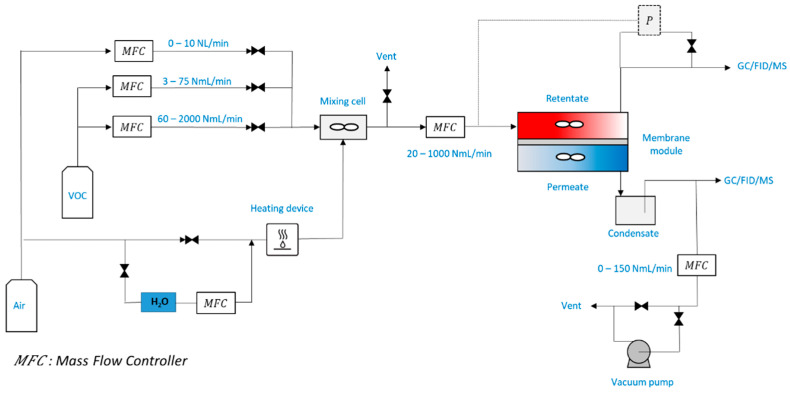
Experimental setup.

The experimental study was carried out with an air-VOC mixture generation system and a flat membrane module (Figure 2). This experimental setup was equipped with a system used to control concentrations, flow rates, pressures, temperature, and relative humidity. The air-VOC mixture was produced using calibrated gas tanks (Air Liquide).

The hybrid device proposed in this experimental study corresponds to a flat membrane module with the implementation of the photocatalyst in the permeate compartment. The photocatalyst (Quartzel^®^-Saint-Gobain Quartz, Courbevoie, France) is made of 10 µm diameter quartz fibers coated with sol-gel TiO_2_-anatase. The corresponding surface density is 120 g m^−2^, including 40 g m^−2^ of TiO_2_. One single layer of photocatalyst had 4 mg cm^−3^ of TiO_2_ per unit volume, i.e., a surface density of TiO_2_ of 4 mg cm^−2^. The medium porosity was close to 0.995. The photocatalytic medium was illuminated by three LEDs (LED Engin—LZ1-00U600—5 W) emitting UV light with a spectral peak centered on 365 nm through borosilicate glass. The volume of the retentate and permeate compartments were 65 mL and 145 mL, respectively. These compartments were considered to be continuous stirred-tank reactors (CSTR).

The first membrane used in this experimental study was a 50 µm thick polydimethylsiloxane (PDMS) rubbery polymeric membrane without support (Goodfellow^®^, Quebec, Canada). PDMS membranes are well suited for the VOC separation from N_2_ to O_2_ or air [33,34,35,36]. The second membrane was a rubbery polymer thin-film composite membrane, with the polyethylene oxide containing block copolymer PolyActive as the selective layer applied on a gutter layer, a PDMS protective layer and a porous support of polyacrylonitrile (PAN) [37]. The PolyActive membrane provides permeabilities that efficiently separate many VOCs from air [38]. A sweep gas flow rate can also feed the permeate compartment to improve the mass transfer through the membrane and dilute possible oxidation products.

The analysis of VOCs in the retentate and permeate fluxes was performed online by a PerkinElmer system coupled with a thermodesorber (TurboMatrix 100, Taman Jurong, Singapore) and a gas chromatography system (Clarus 580, Washington, DC, USA.) equipped with a flame ionization detector and a quadrupole mass spectrometer (Clarus SQ8, Bridgeport Avenue Shelton, CT, USA), respectively. The mineralization rate of toluene was assessed by measuring the CO_2_ concentration in the outflow of both the permeate and retentate compartments by gas chromatography and a pulsed discharge ionization detector. More specifically, the concentration of formaldehyde was monitored in real time with a proton transfer reaction mass spectrometer (Ionicon, Innsbruck, Austria). UVA irradiance was measured with a light detector (Gigahertz Optik radiometer model X11-XD-9511, range 315/400 nm, Washington, DC, USA).

The photocatalytic medium was also analyzed to determine and quantify the presence of possible oxidation products on the surface, such as those described in the literature [25,26,27,28,29]. The analysis was performed primarily with the aim of identifying and quantifying aldehydes and other compounds.

The quantitative analysis of aldehydes and ketones present on the medium surface was performed by desorbing about 200 mg of catalyst in 4 mL of a solution of 2,4-dinitrophenylhydrazine (DNPH)/acetonitrile at 0.34 g mL^−1^. The desorbate was then filtered and analyzed by HPLC coupled to a UV detector, wavelength 360 nm (Shimadzu LC-2010C HT, SpectraLab Scientific Inc. Markham, ON, Canada). The other compounds were analyzed by the desorption of 100 mg of catalyst in 4 mL of carbon disulfide. The extract was filtered and analyzed by gas chromatography-mass spectrometry (Shimadzu GCMS-QP2010 Plus, SpectraLab Scientific Inc. Markham, ON, Canada). The detailed method is described in Supplementary Material.

The operating conditions of the experiments were:

- Membrane diameter 70 mm (membrane surface area = 38.5 cm^2^);
- VOC inlet concentration: 2 and 10 ppm of toluene;
- Feed flow rate: 100 NmL min^−1^, i.e., 7.42 × 10^−5^ mol s^−1^;
- Pressure (*p*″: permeate pressure/*p*′: retentate pressure): 0.98 bar/1.3 bar–0.98 bar/2 bar;
- Sweep gas flow rate: 0%, 10% and 30% of the feed flow rate value;
- Temperature: 24 °C;
- Relative humidity: 1% and 50% (in the feed flow);
- Irradiance: 0.04, 0.3, 1.2, 2.8, 7 and 11 W m^−2^;
- Catalyst mass: *m*0 = 0.02, *m*1 = 0.15 and *m*2 = 0.41 g.

**Figure 3:**
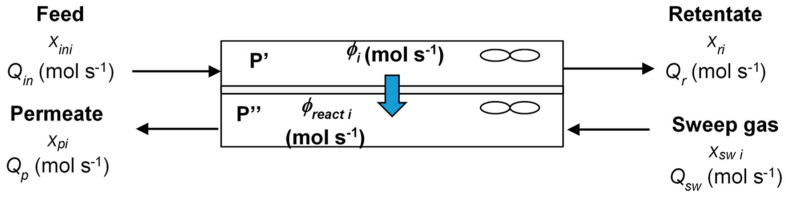
Schematic representation of the experimental module for the molar balance.

The hybrid system presented in Figure 3 is characterized by the following parameters:

- The pressure ratio represents the ratio of the pressures on the permeate and retentate sides:

(1) $$\psi =\frac{P^{\prime \prime }}{P^{\prime }}$$

- The stage cut (%) is the ratio of the permeate flow rate to the feed flow rate:

(2) $$\theta =\frac{Q_{p}}{Q_{in}}\times 100$$

- The recovery ratio of toluene (%):

(3) $$R_{tol}=\left(1-\frac{Q_{r}x_{r_{tol}}}{Q_{in}x_{in_{tol}}}\right)\times 100$$

*x_ini_, x_ri_ x_pi_*_,_ and *x_swi_* correspond respectively to the mole fractions of compound *i* in feed, retentate and permeate compartments, and in sweep gas flow. *Q_in_, Q_r_*, and *Q_p_* are the molar fluxes in feed, on the retentate, and permeate side (mol s^−1^), respectively, *Q_sw_* is the sweep gas flux (mol s^−1^). *φ_i_* is the flux of compound *i* through the membrane (mol s^−1^) and *φ_react i_* is the reaction flux of compound *i* (mol s^−1^).

The mineralization rate of toluene by PCO is defined by Equations (4), (5) and Equation (S1):

(4) $$R_{{CO}_{2}}=\frac{\emptyset_{CO_{2}}}{7\times \emptyset_{reac\;toluene}}\times 100=\frac{\emptyset_{CO_{2}}}{7\times r}\times 100$$

where

(5) $$\emptyset_{CO_{2}}=Q_{r}\times x_{r\;{CO}_{2}}+Q_{p}\times x_{p\;{CO}_{2}}-\left(Q_{in}\times x_{in\;{CO}_{2}}+Q_{sw}\times x_{sw\;{CO}_{2}}\right)$$

### 2.2. Modeling of Toluene Removal in the Experimental Module

The modeling of toluene removal in the experimental system was carried out for an effluent composed of *n* = 5 compounds, i.e., nitrogen, oxygen, toluene, water, and carbon dioxide. The photocatalytic reaction is considered in the permeate compartment. The kinetic model applied for the PCO of toluene is often a Langmuir–Hinshelwood monomolecular model [25,26,27,28,29]. The Langmuir–Hinshelwood model proposed here integrates the light intensity absorbed by the catalyst and the mass of the photocatalytic medium [29,39].

The chemical and mass transfer processes were modeled based on the molar balances and the expressions of the molar fluxes through the membrane. The details of the modeling approach are presented and described in Supplementary Material.

## 3. Results and Discussion

### 3.1. Toluene Removal with the Experimental Hybrid Module

**Figure 4:**
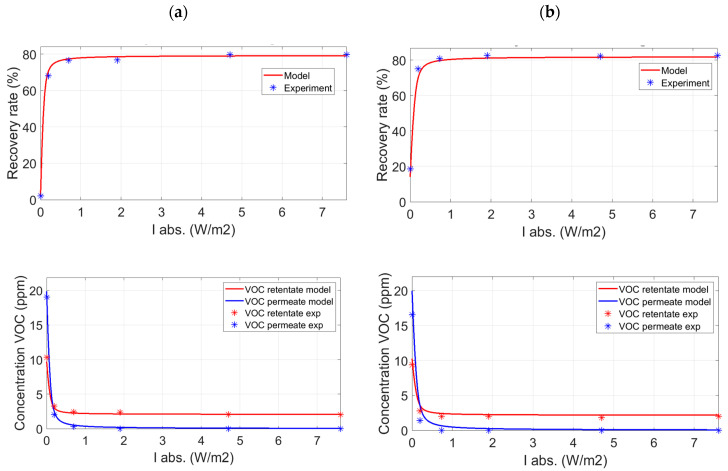
Toluene recovery ratio and toluene retentate/permeate concentrations vs. *I_abs_* for the PDMS membrane (**a**) and the PolyActive membrane (**b**)—catalyst *m*2 − ψ=0.98/2—without sweep gas.

The experimental results presented in Figure 4 represent the evolution of the toluene recovery ratio and the corresponding concentrations as a function of the light intensity absorbed by the catalyst located in the permeate compartment. These experiments were performed without a sweep gas flow rate, i.e., *Q_sw_* = 0 mol s^−1^. For *ψ* = 0.98/2 and a catalyst mass *m2*, the toluene recovery ratio reached a maximum value of about 80% for both the PDMS and the PolyActive membrane. This maximum value was reached and remained independent of *I_abs_* from an absorbed light intensity of about 0.7W m^−2^, corresponding to the transition from the chemical regime to the diffusional regime, as demonstrated in the work of Gérardin et al. [32]. In other words, beyond a certain value of light intensity, the limiting process is the mass transfer through the membrane. The toluene recovery ratio could be significantly improved either by increasing the membrane surface or by decreasing the membrane thickness [33,40].

The PCO of toluene that occurs in the permeate compartment increases the toluene recovery ratio, making it possible to obtain an enhancement factor (*EF*) defined by Equation (6), for the PDMS and PolyActive membranes of 70 and 4.7, respectively. The behavior of the two membranes also differs in the stage cuts, i.e., *θ* = 1% for the PDMS membrane and *θ* = 7.8% for the PolyActive membrane (Table 2). Furthermore, the evolution of the toluene concentration at the outlet of the permeate compartment as a function of *I_abs_* quickly reaches very low values for both types of membranes (Figure 4). The influence of pressure on the retentate side on the toluene recovery ratio was studied at a pressure of 1.3 bar (Figure S3 of Supplementary Material). Equations (S7) and (S11) in Supplementary Material show that the higher the pressure at the retentate (P′), the higher the VOC flux through the membrane will be, improving separation efficiency. In this case, logically, the recovery ratio was lower than that obtained with a pressure of 2 bar and reached a maximum value of 68%. On the other hand, *EF* was significantly increased with a maximum value of 120.

(6) $$EF=\frac{R_{tol\;Max}}{R_{tol\;0}}$$

with *R_tol Max_* for the maximum recovery ratio of toluene (%) and *R_tol_*_0_ for the recovery ratio of toluene (%) with *I_abs_* = 0 W m^−2^.

The coupling of membrane separation and PCO of toluene was modeled based on works detailed in Supplementary Material [32]. The constants of the Langmuir–Hinshelwood type kinetic law proposed in this paper (Equation (S2)) and the overall mass transfer coefficients for each compound were determined experimentally (Table S2 in Supplementary Material). The overall mass transfer coefficients were obtained experimentally when the hybrid system operated in diffusional mode, i.e., when the limiting process was the mass transfer through the membrane and not the chemical reaction in the permeate compartment. Thus, the constants of the kinetic model are *n_i_* = 0.85, *k* = 10^−5^ mol m^−3^ (W m^−2^)^−0.85^ g^−1^ s^−1^, *K* = 1.1 × 10^4^ m^3^ mol^−1^ and the absorption coefficient (Equation (S3)) for the photocatalyst is *α* = 76 m^−1^. The photocatalytic media used in the study had a mass of *m*0 = 0.02 g, *m*1 = 0.15 g, and *m*2 = 0.41 g and a thickness *l* of 0.003, 0.005 m, and 0.015 m respectively.

**Figure 5:**
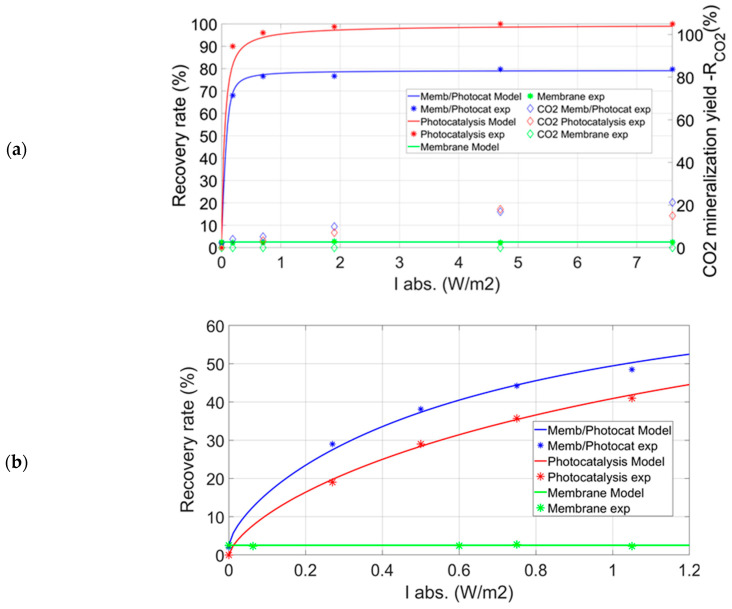
Toluene recovery ratio for three different configurations vs. *I_abs_* − *ψ* = 0.98/2 (**a**) *C_in_* = 10 ppm—*Catalyst m*2 (**b**) *C_in_* = 2 ppm—Catalyst *m*0*—*without sweep gas.

The model used makes it possible to make relatively precise predictions for the recovery ratio of toluene, the concentrations of the different compounds (Figure 4 and Figure 5), and the stage cuts according to the operating conditions. The model of the evolution of TPs was not investigated in this study, in particular because of the difficulty of accessing the corresponding kinetics.

The influence of relative humidity on the different processes in the study was assessed (Figure S4). The results show the toluene recovery ratio obtained by PCO and by the hybrid process for relative humidities of 1% and 50% with *I_abs_* = 6.9 w m^−2^, and a catalyst mass *m*2 = 0.41 g. For all the experimental configurations presented in this paper, the influence of relative humidity on the toluene removal rate was weak. This clearly shows the robustness of the hybrid process to humidity, unlike the VOC adsorption treatment, for example. Moreover, the temperature increase in the photocatalytic reactor and in the hybrid module related to the chemical reaction and to the heating of the LEDs was about 6 °C, i.e., a temperature of 30 °C in the experimental systems.

The experiments were conducted to assess the toluene recovery ratio vs. *I_abs_* for processes implemented separately or coupled. Figure 5a presents the toluene recovery ratio for the three different configurations, i.e.,

- Photocatalytic decomposition in the experimental module without membrane (CSTR), catalyst mass *m*2. The modeling of PCO reactor is detailed in Supplementary Material.
- Membrane separation of toluene without PCO and with the same pressure ratio.
- Coupling membrane separation and PCO in the permeate compartment of the experimental module, catalyst mass *m*2, PDMS membrane, without sweep gas flow rate.

Under these operating conditions, the PCO of toluene alone leads to a higher recovery ratio than when coupling occurs. PCO alone allows reaching a high decomposition rate close to 100%. On the other hand, the membrane-photocatalysis coupling shows a toluene removal rate of about 80%. The difference in efficiency observed between the two processes is due to the limiting operating regime of the hybrid configuration, i.e., mass transfer through the membrane [32]. When the chemical kinetics of toluene decomposition is slowed down because of a lower catalyst mass and/or the light intensity absorbed by the catalyst, the membrane-PCO coupling becomes more advantageous than the PCO alone (Figure 5b). Thus, for a catalyst mass *m*0 = 0.02 g and without sweep gas, the hybrid process provides higher performances than those obtained with the PCO. Moreover, it is quite possible to set up a thinner membrane, a larger surface area, sweep gas, or higher pressure and thus make the hybrid process perform even better.

On the other hand, membrane separation alone enables only very low recovery of toluene, regardless of the light intensity used. Figure 5a also shows the toluene mineralization rate as a function of *I_abs_* for the three experimental configurations. The highest toluene mineralization rate, *R_CO_*_2_, is around 20% for the membrane-PCO coupling and for *I_abs_* = 11 W m^−2^. Overall, the hybrid configuration seems to lead to a slightly higher mineralization rate of toluene than PCO alone, but this is not very significant here.

### 3.2. Identification and Monitoring of the Formation of Transformation Products

The research and identification of toluene oxidation products were carried out on the photocatalytic medium surface at the end of the experiment, as well as in the effluents from the retentate and permeate compartments of the module. The results of analyzing the different media are presented in Table 3. The experiments were conducted in a configuration with photocatalysis alone and in a hybrid membrane-PCO configuration with *m*2 photocatalysts over 13 h. The main compounds identified on the media surface were aldehydes and ketones. These compounds were quantified but the other organic compounds identified at trace level were not quantified.

The results presented in Table 3 show that among the different aldehydes and ketones identified on the medium surface, benzaldehyde is the major transformation product on the photocatalyst. It appears that the formation of benzaldehyde could be favored in the configuration with photocatalysis alone compared to the hybrid mode for an equivalent mass of decomposed toluene. This process was also observed for the other compounds, except acetone. According to the studies available in the literature, benzaldehyde appears to be the main compound responsible for the deactivation of the catalyst [25].

The main organic TPs identified in trace amounts on the catalysts after the experiment are as follows: o-xylene, styrene, benzyl alcohol. On the fresh catalyst used as a reference, the following were observed: toluene, o-xylene, styrene, 2-butanone.

In the gas phase, the compounds observed in the different experimental configurations in the outlet effluents were formaldehyde, acetaldehyde, benzaldehyde, acetone, and benzene. Formaldehyde, acetone, and especially benzene are transformation products that appear gradually over time. As such, and because of their high toxicity for humans [4], specific monitoring of the evolution of benzene and formaldehyde concentrations in the effluents according to the mass of toluene eliminated was carried out for the experimental configurations under investigation.

**Figure 6:**
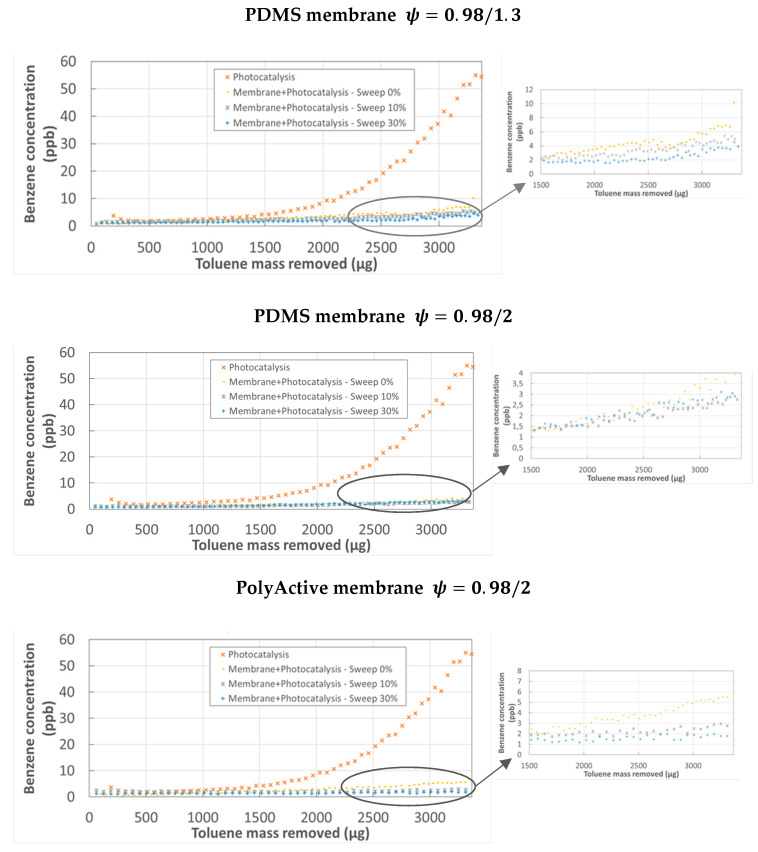
Evolution of benzene concentration in the retentate flow vs. toluene mass removed—catalyst *m*2$-I_{abs}=6.9\;W\;m^{-2}$.

Figure 6 represents the evolution of the benzene concentration as a function of the mass of toluene removed in the case of photocatalysis alone and in the case of the hybrid process of membrane separation-PCO. It is clear that PCO alone of toluene progressively leads to the formation of benzene, the concentration of which can reach 60 ppb in the reactor outlet flow. On the other hand, the combination of membrane separation and PCO considerably limits the presence of benzene in the effluent from the retentate compartment. Thus, for the cases of the PDMS and PolyActive membrane with the *m*2 catalyst, the implementation of a sweep gas in the permeate compartment with a flow rate equal to 30% of the feed flow rate and a pressure couple = 0.98/2 leads to a slight increase of the benzene concentration, amounting to 0.5 ppb. The coupling thus enabled a reduction of the presence of benzene by a factor higher than 120. For these operating conditions, the benzene concentrations at the outlet of the permeate compartment were 6 and 8 ppb respectively for the PDMS and PolyActive membranes. The limitation of benzene in the retentate compartment of the module had several possible causes. The membrane-PCO coupling led to a higher residence time in the volume in which oxidation occurs than in the simple photocatalytic reactor. This led to greater progression of the different reactions and to lower TP production. The presence of a membrane and a pressure ratio *ψ* = 0.98/2 facilitated the trapping of the TPs formed in the permeate compartment (trap effect). This trapping effect is as efficient as the permeability of the membrane relative to benzene is low. Increasing the thickness of the membrane or reducing its surface area should also help to intensify the trapping of undesirable TPs in the permeate compartment. However, from the perspective of hybrid module design, further study will be required to determine the optimal values for key parameters such as membrane thickness and surface area. The goal will be to propose the best thickness/surface area configuration in order to obtain the highest pollutant transfer to the permeate compartment with the lowest flux of TPs to the retentate compartment.

Finally, the sweep gas in the permeate compartment of the system, which can reach 30% of the feed flow, reduced the concentration of these compounds by dilution and reduced the corresponding driving force between the two compartments of the system. As a result, the presence of benzene in the effluent in the outflow of the retentate compartment was limited at a reduced energy cost.

**Figure 7:**
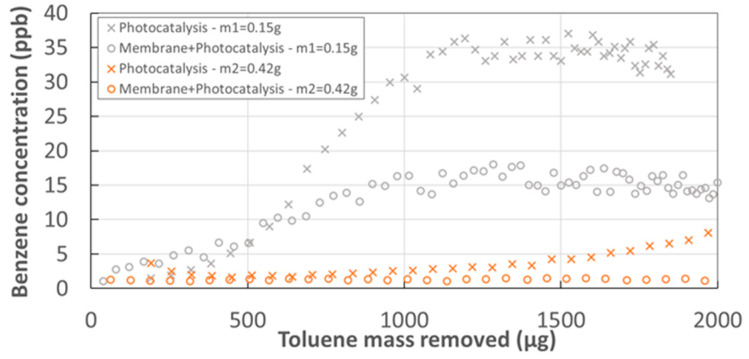
Evolution of benzene concentration in the retentate flow vs. toluene mass removed for catalysts *m*1 and *m*2*—*PDMS membrane—Sweep gas = 0*%*$-I_{abs}=6.9\;{W\;m}^{-2}-\psi =0.98/2$.

**Figure 8:**
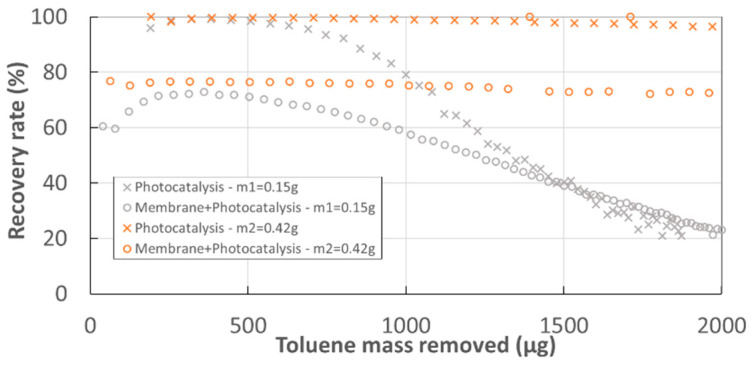
Toluene recovery ratio vs. toluene mass removed for catalysts *m*1 and *m*2—PDMS membrane Sweep gas = 0%$-I_{abs}=6.9\;{W\;m}^{-2}-\psi =0.98/2$.

One important parameter in the process was the catalyst mass implemented. Figure 7 shows that for a lower catalyst mass (*m*1 = 0.15 g), benzene formation appears almost from the beginning of the experiment and benzene concentrations are significantly higher. However, the membrane-PCO coupling greatly reduces the presence of benzene in the retentate compartment. This benzene production is correlated with the loss of photocatalyst activity (Figure 8). It appears that the loss of activity of the catalyst is as rapid as the mass implemented is low. This phenomenon was observed for both PCO alone and for the hybrid configuration. However, the loss of activity of the catalyst seemed to be less accentuated in the case of the hybrid setup, in particular for the *m*1 catalyst compared to PCO alone.

**Figure 9:**
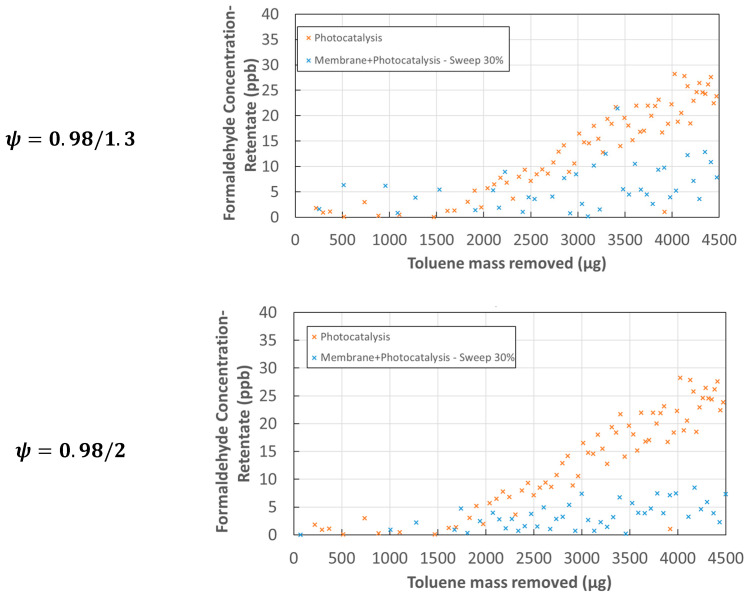
Evolution of formaldehyde concentration in the retentate flow vs. toluene mass removed—PDMS membrane—catalyst *m*2*—*Sweep gas = 30% $-I_{abs}=6.9\;{W\;m}^{-2}$.

With regard to formaldehyde, the concentration in the outlet effluents was monitored for the different configurations as a function of the mass of toluene removed. Figure 9 shows the results for PCO alone of toluene and for the hybrid configuration. All the tests were conducted with an absorbed light intensity of 6.9W m^−2^ by the *m*2 catalyst and with a 30% sweep gas. PCO alone led to the formation of formaldehyde from 1500 µg of decomposed toluene and the concentration reached about 25 ppb at the end of the experiment. In the case of the membrane-PCO association, the appearance of formaldehyde was much less considerable than in the previous case and the presence of formaldehyde in the retentate stream was around 10 ppb and 5 ppb for a pressure ratio *ψ* of 0.98/1.3 and 0.98/2 respectively. Although this is less significant than for benzene, the hybrid configuration contributed to limiting the presence of formaldehyde in an effluent that could be reused. Moreover, although parameter *ψ* has a strong influence on the trapping effect of the system, the nature of the membrane material, its surface, its thickness, and its implementation were decisive parameters for accentuating and increasing the capacity of the hybrid device to limit the presence of TPs in the retentate side.

**Figure 10:**
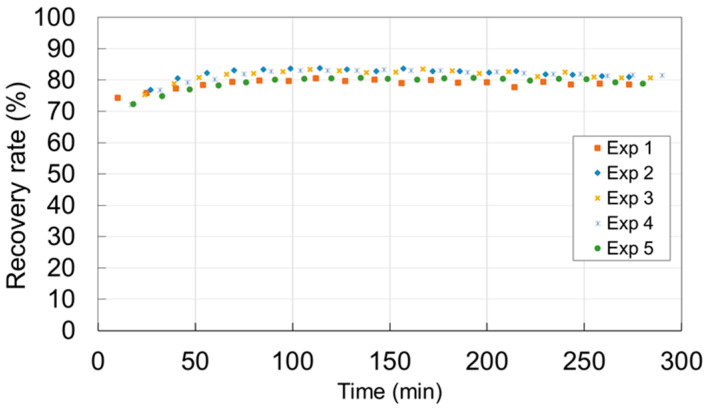
Comparison of toluene recovery ratio vs. time for different experiment cycles—catalyst *m*2—ψ=0.98/2.

The formation of TPs seems to be related to the loss of catalyst activity (Figure 7, Figure 8 and Figure 9). Previous work has shown that the catalyst deactivated during the toluene oxidation process can be regenerated [13]. The study presented in this paper demonstrated that it is possible to regenerate both *m*1 and *m*2 catalysts. For example, Figure 10 shows that the *m*2 catalyst can be regenerated at least five times and its initial activity after being completely deactivated is restored. The regeneration consists in exposing the catalyst to UV-A radiation with an irradiance of 70 W m^−2^ for 15 h in ambient air with a relative humidity between 20% and 50%. The analysis of the regenerated catalysts by the analytical method described above revealed the presence of very low traces of benzaldehyde and other TPs.

## 4. Conclusions

Whether in a domestic or occupational context, the challenge of limiting the exposure of various populations to toxic compounds has led science and technology to propose systems designed to improve the quality of atmospheres. Photocatalytic oxidation has quickly generated strong interest among engineers. However, the technological development of this oxidative technique has had to address the question of the formation of TPs. This threat is clearly the main barrier to the widespread application of photocatalytic oxidation for VOC removal. The study presented in this paper demonstrated that the original integration of a photocatalytic process in the permeate compartment of a membrane separation module makes it possible to significantly reduce the presence of TPs in the outflow effluent.

The demonstration carried out with toluene, in which the formation of photocatalytic oxidation products is well-known, highlighted that membrane separation-PCO hybridization can be slightly more efficient than PCO alone in terms of VOC removal efficiency. However, it is particularly important to simultaneously reduce the presence of benzene and formaldehyde in the effluent to so that the catalyst can be reused. Benzene and formaldehyde are compounds that are carcinogenic for humans and their concentration must be kept as low as possible in the breathable air. Given its low flow rate, a post-treatment of the permeate by PCO or other advanced oxidation processes could also be considered before its release into the atmosphere. The implementation of different experimental conditions revealed that it is possible to reduce the deactivation of the catalyst. This hybrid process also has other advantages, including the option of working with a solar power source and limiting the production of waste. Furthermore, the process presented here does not require high pressure in the retentate compartment, as the driving force for any separative process is provided by chemical pumping in the permeate compartment. Engineers who intend to design a VOC treatment device as an alternative to more conventional solutions, such as adsorption or absorption, can use this hybrid “low carbon” process as a toolbox to make photocatalytic oxidation safer. The modeling of membrane separation-PCO coupling, based on experiments, highlighted the various operating parameters that can be used to propose efficient technological solutions. Although it will be useful to consolidate this work with the study of other compounds, the applications of this coupling for the purification of industrial or medical gases and for the cleaning of indoor air are numerous.

## Figures and Tables

**Table 1:** Typical concentrations of toluene, benzene, and formaldehyde observed in indoor air.

	Household (ppb)	Workplace (ppm)
Toluene	1–45 [7]	0.001–1000 [7,9]
Benzene	0.5–15 [10]	0.002–1.1 [10]
Formaldehyde	7–220 [7,11]	0.04–0.8 [7,11]

**Table 2:** Experimental and modeled stage cuts for different values of *ψ*-.

Membrane	*ψ*	*θ_exp_* (%)	*θ_mod_* (%)
**PDMS**	**0.98/1.3**	0.5	0.4
	**0.98/2**	1.0	1.2
**PolyActive**	**0.98/1.3**	3.0	2.4
	**0.98/2**	7.8	8.0

**Table 3:** Mass concentrations of the main aldehydes and ketones identified on the photocatalytic media (µg/g).

	PCO	Membrane + PCO	Fresh Catalyst
**Formaldehyde**	7.9	6.1	5.1
**Acetaldehyde**	13	9.3	16.2
**Acrolein**	0.3	0.3	0
**Acetone**	46	103	32
**Propionaldehyde**	0.4	0.2	0.1
**Butanal**	1.9	1.1	0.1
**Benzaldehyde**	**1460**	**964**	**1.7**
**Pentanal**	1.4	1.1	0.2
**m-Tolualdehyde**	0.8	0.6	0.7
**Hexanal**	0.7	0.4	0.2

## Data Availability

Not applicable.

## Supplementary Materials

The following supporting information can be downloaded at: https://www.mdpi.com/article/10.3390/membranes12090900/s1, Figure S1: Schematic representation of the experimental module for molar balance; Figure S2: Representation of the different layers of the membrane system and the corresponding resistances; Figure S3: Toluene recovery ratio vs *I_ab_s* for PDMS membrane − catalyst *m*2 − *ψ* = 0.98/1.3.; Figure S4: Toluene recovery ratio vs Relative Humidity (RH) *– I_abs_* = 6.9 W m^−2^ – PDMS membrane − catalyst *m2*; Table S1: Limits of detection (LOD) and limits of quantification (LOQ) for aldehydes and ketones identified on the medium surface. Table S2: Overall mass transfer coefficients of compounds for the PDMS and PolyActive membranes.
