# Supplementary material for: Experimental Proof of a Transformation Product Trap Effect with a Membrane Photocatalytic Process for VOC Removal
Source: Membranes (Basel). 2022 Sep 19;12(9):900. doi: 10.3390/membranes12090900 (PMC9502665; doi:10.3390/membranes12090900)
Supplement: Supplementary file 1 [file membranes-12-00900-s001.zip › membranes-1898874-supplementary.pdf]

Supplementary material

1. *Analytical method for identification and quantification of transformation products on surface medium*

1.1 Quantitative HPLC/UV analysis of Aldehydes and ketones

A fraction of the 4 samples of photocatalytic medium (200 mg) is extracted in 4 mL of a DNPH/acetonitrile solution at 0.34g/mL. The extract is then filtered for HPLC/UV analysis under the following conditions:

- Injection volume: 10 µL

- Column: Restek C18, 25cm, 4.6 mm, 5 µm

- Eluent flow rate: 1 mL/min

- Eluent: Water/acetonitrile (40/60)

- Analysis time: 20 minutes

- Column temperature: 30°C

- UV wavelength: 360 nm

Limits of detection (LOD) and limits of quantification (LOQ) for aldehydes and ketones identified on the medium surface are presented Table S1.

1.2 Qualitative analysis in GC/MS pf other compounds

Approximately 100 mg of catalysts are extracted into 4 mL of carbon disulfide. The extract is filtered and analyzed in GC/MS under the following conditions:

- Column: vf-624-ms, 60m, 0.32 mm, 1.8µm

- Column flow rate: 1.8 mL/min

- Injection temperature: 270°C

- Split ratio: 15

- Transfer line temperature: 200°C

- Source temperature: 230°C

- Acquisition parameters : 30 < m/z< 550

	LOD	LOQ	LOD	LOQ	**LOD (µg/g)**	**LOQ (µg/g)**
	**ng/mL of desorbat**		**ng**		**µg/g of catalyst**	
**Formaldehyde**	0,0049	0,0163	0,0196	0,0652	0,0001	0,0003
**Acetaldehyde**	0,0077	0,0256	0,0307	0,1024	0,0002	0,0005
**Acrolein**	0,0093	0,0309	0,0371	0,1235	0,0002	0,0006
**Acetone**	0,0106	0,0354	0,0425	0,1418	0,0002	0,0007
**Propionaldehyde**	0,0124	0,0414	0,0497	0,1657	0,0002	0,0008
**Butanal**	0,0199	0,0663	0,0796	0,2654	0,0004	0,0013
**Benzaldehyde**	0,0269	0,0897	0,1076	0,3587	0,0005	0,0018
**Pentanal**	0,0418	0,1394	0,1673	0,5578	0,0008	0,0028
**m-Tolualdehyde**	0,0361	0,1202	0,1442	0,4808	0,0007	0,0024

Table S1 Limits of detection (LOD) and limits of quantification (LOQ) for aldehydes and ketones identified on the medium surface

2. Modeling of the experimental module

This section is dedicated to the modeling of the experimental system (figure S1) for an effluent composed of n=5 compounds: nitrogen, oxygen, toluene, water and carbon dioxide. The photocatalytic reaction is considered in the permeate compartment. The following assumptions are made:

- Retentate and permeate compartments are CSTRs;

- Constant permeability for each compound;

- Steady state of the various phenomena;

- Isothermal conditions of the studied system;

- Uniform thickness of the membrane;

- Constant pressure in each compartment of the module.

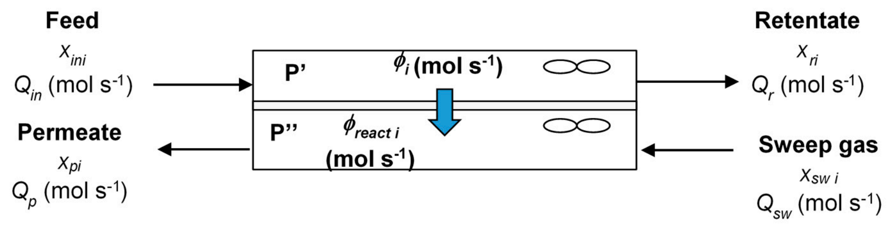

Figure S1. Schematic representation of experimental module for molar balance

x_{ini}, x_{ri} x_{pi} and x_{swi} correspond respectively to the mole fractions of compound *i* in feed, retentate and permeate compartments and in sweep gas flow. Q_{in}, Q_r and Q_p are the molar fluxes in feed, on the retentate and permeate side (mol s^{-1}), respectively, Q_{sw} is the sweep gas flux (mol s^{-1}). ϕ_i is the flux of compound *i* through the membrane (mol s^{-1}) and $\phi_{react\ i}$ is the reaction rate of compound *i* (mol s^{-1}).

The different processes are modeled based on the molar balances and the expressions of the molar fluxes through the membrane.

Photocatalytic Decomposition of Toluene

The production of highly oxidative OH• radicals by the light activation of TiO_2 leads to the PCO of toluene according to the equation for a complete mineralization (S.1) [21–25]:

$$C_7H_8 + 9O_2 \xrightarrow{TiO_2, h\nu} 7CO_2 + 4H_2O \quad \text{(S.1)}$$

Before a complete mineralization of toluene, many intermediate compounds can appear during the photocatalytic process. The kinetic model applied for the PCO of toluene is often a Langmuir-Hinshelwood monomolecular model. In this paper, the light intensity absorbed by the catalyst and the mass of the photocatalytic medium are integrated [26,36]:

$$r = mI_{abs}^{n_i} \frac{kKC}{1+KC} = mI_{abs}^{n_i} \frac{kKx(\frac{P}{RT})}{1+Kx(\frac{P}{RT})} \quad \text{(S.2)}$$

with *r* for the reaction rate (mol s^{-1}), *m* for the mass of photocatalytic medium (g), I_{abs} for the absorbed light intensity by the photocatalytic medium ((Wm^{-2})^{ni}, n_i for the order with respect to I_{abs}, *k* for the apparent kinetic constant (mol m^{-3} (W m^{-2})$^{-ni}$ g^{-1} s^{-1}), *K* for the adsorption constant (m^3 mol^{-1}) and *C* for the concentration of toluene in the reactor (mol m^{-3}). The reaction rate is also expressed with *x* as the mole fraction of toluene, *R* as the perfect gas constant (J mol^{-1} K^{-1}), *T* as the temperature (K) and *P* as

the pressure (Pa). The kinetic study was conducted with a CSTR reactor. The light intensity absorbed by the photocatalytic medium was measured as a function of the thickness of the medium [29]. The light absorption model is:

$$I_{abs} = I_0\left(1 - e^{(-\alpha l)}\right) \qquad \text{(S.3)}$$

with I_0 for the irradiance received by the photocatalytic medium (W m^{-2}), α for the absorption coefficient (m^{-1}) and l for the thickness of the photocatalytic medium (m).

Overall molar balance

$$Q_{in} = Q_r + Q_p - \sum_{i=1}^{n} \phi_{react\ i} \quad \text{(S.4)}$$

Overall molar balance for each compound i

$$Q_{in} x_{in\ i} = Q_r x_{ri} + Q_p x_{pi} - \phi_{react\ i} \qquad \text{(S.5)}$$

And

$$\sum_{i=1}^{n} x_{ri} = 1 \qquad \text{(S.6)}$$

Molar flux through the membrane for each compound *I* without a boundary layers or in the presence of a negligible boundary layers

$$\phi_i = \frac{SP_i}{z}\left[P' x_{r\ i} - P'' x_{pi}\right] \qquad \text{(S.7)}$$

And

$$x_{p\ i} = \frac{\phi_i - \phi_{react\ i}}{\sum_{i=1}^{n}(\phi_i - \phi_{react\ i})} \qquad \text{(S.8)}$$

$$\sum_{i=1}^{n} x_{pi} = 1 \qquad \text{(S.9)}$$

$$\phi_{react\ i} = \nu_i r \qquad \text{(S.10)}$$

$\phi_{react\ i}$ can be positive or negative depending on whether it is a compound that appears or disappears. S and z are the surface area (m^2) and thickness (m) of the membrane, respectively. Pi is the membrane permeability of the compound i (Barrer or mol m^{-1} s^{-1} Pa^{-1}). ν_i is the stoichiometric coefficient of compound i from equation (S.1). ν_i is positive for products and negative for reactants.

ϕ_i is expressed by taking into account the different resistances, in particular those of the laminar mass transfer boundary layers on either side of the membrane (Figure S2):

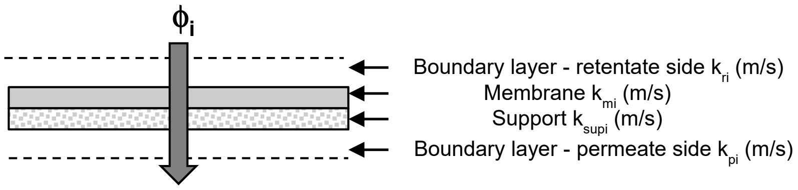

Figure S2 Representation of the different layers of the membrane system and the corresponding resistances

$$\phi_i = K_i S[C_{ri} - C_{pi}] = S \frac{[C_{ri} - C_{pi}]}{\frac{1}{k_{ri}} + \frac{1}{k_{mi}} + \frac{1}{k_{supi}} + \frac{1}{k_{pi}}} \qquad (S.11)$$

$$\frac{1}{K_i} = \frac{1}{k_{ri}} + \frac{1}{k_{mi}} + \frac{1}{k_{supi}} + \frac{1}{k_{pi}} \qquad (S.12)$$

K_i (m s^{-1}) represents the overall mass transfer resistance or overall mass transfer coefficient of compound i through the membrane.

In some cases, for example when the permeability of a compound is very high, a concentration gradient may appear at its boundary layer on the retentate side. This phenomenon, named concentration polarization, results in a decrease in the separation efficiency of the membrane for this compound. It is generally accepted that there is no concentration gradient on the permeate side. However, if a sweep gas is applied on the permeate side or, as in the present case, a reaction takes place in this compartment, it is likely that a concentration gradient will appear in the boundary layer on the permeate side.

3. Modeling of single photocatalytic CSTR

The modeling of a photocatalytic reactor operating in CSTR mode is represented by equation (S.10). This modeling is used to compare the toluene removal performance of this type of reactor with that of a hybrid module described in the previous section.

$$x_{r\_tol} = \frac{Q_{in} \times x_{in_{tol}} - r}{Q_p} \qquad (S.13)$$

With

$$r = m I_{abs}^{n_i} \frac{kKC}{1+KC} = m I_{abs}^{n_i} \frac{kKx(\frac{P}{RT})}{1+Kx(\frac{P}{RT})} \qquad (S.14)$$

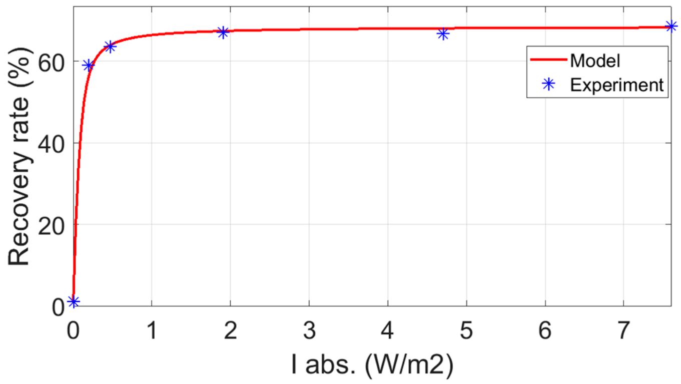

Figure S3. Toluene recovery ratio vs I_{abs} for PDMS membrane - catalyst *m2* -$\psi = 0.98/1.3$

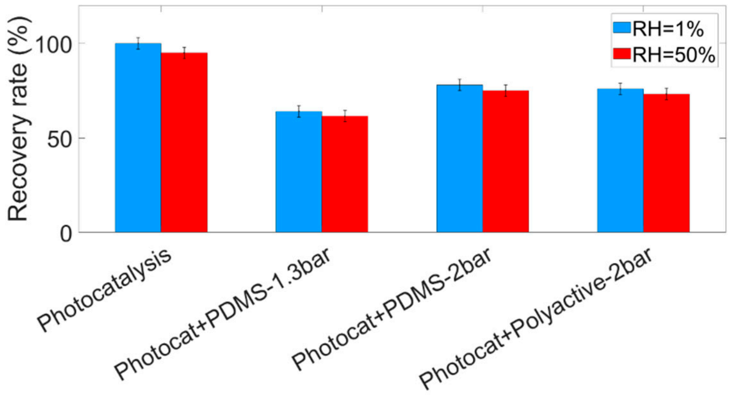

Figure S4. Toluene recovery ratio vs Relative Humidity (RH) - I_{abs} =6.9 W m^{-2} - PDMS membrane - catalyst *m2*

Membrane	K_{tol} (m s^{-1})	K_{N2} (m s^{-1})	K_{O2} (m s^{-1})	K_{CO2} (m s^{-1})	K_{H20} (m s^{-1})
**PDMS**	9x10^{-4}	4.2x10^{-6}	8.4x10^{-6}	5.0x10^{-5}	5.8x10^{-4}
**PolyActive**	9.5x10^{-4}	3.0x10^{-5}	8.1x10^{-5}	-	-

Table S2. Overall mass transfer coefficients of compounds for the PDMS and PolyActive membranes
